# Update of Individualized Treatment Strategies for Postural Orthostatic Tachycardia Syndrome in Children

**DOI:** 10.3389/fneur.2020.00525

**Published:** 2020-06-11

**Authors:** Qingyou Zhang, Bowen Xu, Junbao Du

**Affiliations:** ^1^Department of Pediatrics, Peking University First Hospital, Beijing, China; ^2^Research Unit of Clinical Diagnosis and Treatment of Pediatric Syncope and Cardiovascular Diseases, Chinese Academy of Medical Sciences, Beijing, China; ^3^Key Laboratory of Molecular Cardiovascular Sciences, The Ministry of Education, Beijing, China

**Keywords:** autonomic dysfunction, orthostatic intolerance, postural orthostatic tachycardia syndrome, treatment, children

## Abstract

Postural orthostatic tachycardia syndrome (POTS) is a heterogeneous disease that predominantly affects children and adolescents. There is a great difference between children and adults in the diagnosis and treatment of POTS patients. POTS in children and adolescents is marked by chronic symptoms of orthostatic intolerance with a heart rate (HR) rise of ≥40 bpm, or heart rate exceeding 130 bpm for 6–12-years-old children and exceeding 125 bpm for those 13–18 years old without orthostatic hypotension, which is different from adult patients. The three major clinical forms of POTS include hypovolemic POTS, neuropathic POTS, and hyperadrenergic POTS; these are distinguished by their major mechanisms. The different subtypes of POTS in children and adolescents each have their own clinical characteristics and biomarkers. Based on these, we propose individualized treatment strategies. Individualized management strategies based on different subtypes of POTS would largely improve the curative effects of drugs for children with POTS. However, a further clinical investigation is still required to better understand the pathophysiology and treatment options.

Postural orthostatic tachycardia syndrome (POTS) is a form of chronic orthostatic intolerance (1). POTS is more common in children than in adults, and most POTS patients develop their symptoms in childhood or adolescence (2, 3). It is increasingly recognized in children (4, 5). However, because of the varying symptoms of POTS in children and adolescents, including cardiovascular, neurologic and gastrointestinal symptoms, it is often misdiagnosed. In recent years, more and more attention has been paid to the diagnosis and treatment of this syndrome (6–9). This review details the characteristics of POTS in pediatric patients.

POTS is a syndrome, not a disease, and features hemodynamic abnormalities of upright position and other symptoms. It results in inability to attend school, take part in physical activities and even the normal activities of daily life. As a result, POTS patients face significant social and economic consequences (9–12), and early diagnosis is crucial to the launch of effective therapy. However, the clinical features of POTS in children and adolescents have not yet been fully summarized (13). Therefore, we reviewed the literature related to pediatric POTS, which provides a broad understanding of the characteristics of this syndrome.

## Clinical Characteristic of Pots in Pediatric Patients

POTS is a common disorder of chronic orthostatic intolerance in children. The disorder is characterized by dizziness, palpations, fatigue, headache, chest tightness, abdominal pain, nausea, and even syncope on standing. Although its symptoms are primarily associated with the upright position, some patients report their persistence when they are sitting or lying down. The definition of POTS is that the symptoms of orthostatic intolerance persist at least 6 months, and are accompanied with an increase in heart rate (HR) exceeding 30 bpm (or a rate that exceeds 120 bpm) within 10 min of standing or achieving upright tilt without orthostatic hypotension (>20 mmHg drop in systolic blood pressure) (1–6, 8). However, children and adolescents display different pictures related to their particular types of growth and development. In our previous study, the HR and blood pressure of 1,449 school children (6–18 years old) in China were recorded during active standing. During tests, the HR and BP had significantly changing profiles in children and adolescents, and our group proposed that the diagnosis of POTS in the pediatric population be made when the HR rises by ≥40 beats per minute, or the fastest heart rate of children aged 6–12 exceeded 130 beats per minute, and exceeded 125 beats per minute for adolescents aged 13–18, within 10 min of standing, accompanied by symptoms of orthostatic intolerance (13–15).

POTS predominantly affects females, with the ratio of females to males in adult studies being 4:1 (4, 12, 16). Researchers speculate that menstruation, which features periodic variations in levels of estrogen and progesterone, is related to the incidence of POTS (17). However, in our cohort, the male: female gender ratio was only 1:1.1, since we expected that the impact of menstruation in children would be small (5). POTS can occur throughout adolescence, and the children with POTS were mainly aged between 7 and 14 years in this study, a figure consistent with previous reports from our center (4, 5).

The symptoms of POTS in children and adolescents vary. Common symptoms of our patients include dizziness (84.00%), fatigue (72.00%), orthostatic faint (62.67%), shortness of breath (55.33%), pallor (51.33%), blurred vision (50.00%), hyperhidrosis (43.33%), gastrointestinal difficulties (40.67%), and fatigue (37.33%) (5). Some authors have reported that 30% of their patients have a variety of symptoms (18). Female patients often experience a worsening of symptoms during menstruation.

The numerous co-morbidities of POTS in children have attracted the attention of many researchers. These include chronic fatigue syndrome, sleep disorders, migraines, irritable bowel syndrome or functional dyspepsia, cyclic-vomiting syndrome, fibromyalgia, and Ehlers-Danlos syndrome (6, 18). However, the relationship between these disorders and POTS is still unclear. Many investigators suggest that POTS share the similar mechanisms for the co-morbid disorders. However, some authors have found that no difference can be found in the incidence of those co-morbid conditions in children and adolescents with POTS and those without POTS. They are syndromes which may occur together with POTS, but POTS itself is not a cause of the co-morbidities (19).

Psychiatric disorders including anxiety and depression are associated with POTS in child and adolescent family members of patients, and child patients with POTS often encounter tragic experiences after contracting the illness, with no regular attendance at school and social activities, underachievement, and frequent visits to doctors. They often receive a variety of diagnoses, take multiple drugs, and sometimes even receive surgery.

## Pots Subtypes in Children and Adolescents

Based on the mechanisms of potential pathophysiology of POTS, 3 main clinical subtypes of the syndrome have been established: hypovolemic POTS, neuropathic POTS, and hyperadrenergic POTS (20–22). These phenotypes may overlap.

### Hypovolemic POTS

Central hypovolemia leads to a decrease in venous return, resulting in an increase of heart rate as compensation. Low blood volumes have been observed in many children and adolescents with POTS (23, 24). In patients with hypovolemia, the abnormal activation of the renin-angiotensin-aldosterone system has been found in children (24–26). Hypovolemia could be observed in nearly 30% of POTS patients, as Thieben et al. estimated (16). In our experience, over fifty percent of children and adolescents with POTS have low blood volume (5). Hypovolemic POTS patients have symptoms, such as obvious weakness and decreased tolerance for exercise. Increasing central blood volume with intravenous fluids or salt supplements can significantly improve these patients' symptoms (23, 27). El-Sayed and Hainsworth found that 24-h urinary sodium was a valuable marker of hypovolemic status (23). According to our research, a 24-h sodium excretion of <124 mmol is a good indicator of the effectiveness of replenishment of blood with salt supplementation in children and adolescents with POTS (27).

### Neuropathic POTS

Partial autonomic neuropathy is the characteristics of this subtype of POTS. The main mechanism of this type of POTS is patchy denervation of the sympathetic fibers to the blood vessels in the extremities (28). We found that the incidence of Valsalva maneuver (Valsalva ratio, VR) of <1.5 was 84.72% in our cohort patients with POTS (5). In another study, loss of sweating function of the extremities was found in over 50% of patients with POTS (16). The denervation of the sympathetic fibers to the blood vessels in the extremities might affect their contractile function and cause the pooling of blood in the extremities. When patients stand up, their abnormal vessel tone and pooling in the lower extremities lead to a decrease of returned-blood volume, causing a decrease of cardiac output, and a heart-rate increase in compensation. We also found that some patients with POTS had augmented flow-mediated vasodilation (FMD) of the brachial artery, indicating that an increase of the vasodilation response of the peripheral may lead to the poor blood circulation of the lower extremities when standing, resulting in a decrease of returned-blood volume and symptoms of POTS (29). Severe venous pooling in the lower extremities is characterized by cyanosis of the feet upon standing in this subtype of POTS children. Many patients report having a history of fever before the onset of disease, likely viral infection, or having a history of surgery, infection or trauma. Therefore, some researchers propose immune pathogenesis of neuropathic POTS (30). We found that 24.39% of patients were positive for antibodies of acetylcholine receptor (AChR-ab). The symptoms of those POTS children were significantly severe and syncope and fatigue were common (31). Other autoimmune antibodies in POTS, such as alpha 1 and/or beta adrenergic-receptor autoimmune antibodies, and angiotensin II Type 1-receptor autoimmune antibodies, were also observed (32, 33). At present, it is considered that neuropathic POTS is a mild autoimmune disorder.

### Hyperadrenergic POTS

The definition of hyperadrenergic POTS is a syndrome with an increase of above 10 mmHg in systolic BP within 10 min of standing or tilting, and an upright-position plasma norepinephrin of ≥600 pg/mL (34, 35). In our cohort, 51.28% of children with POTS were hyperadrenergic. These patients manifested with hypertension upon standing, and may complain of lightheadedness, faintness, palpitations, shortness of breath, syncope, tremulousness, headache, fatigue and nausea and vomiting. The most common symptoms of hyperadrenergic POTS in children are dizziness, headache and tremulousness, compared with other types of POTS patients (35). Hyperadrenergic POTS can be caused by norepinephrine transporter deficiency (36), pheochromocytoma, mast-cell-activation disorders (37), and baroreflex failure resulting from trauma to or irradiation of the neck. However, most of the children had not suffered either of these.

## Treatment Approaches (Table 1)

Accurate diagnosis is the basis for controlling this disease that excludes true cardiac disorders. The majority of patients with POTS show substantial improvement after proper diagnosis leads to a comprehensive therapeutic regimen being put in place. There are many ways to treat the disease, including drugs and physical therapy.

**Table 1:** The main clinical studies included in the review.

**Study group**	**Methods**	**No. of patients**	**Drug**	**Biomarkers**	**Outcomes**
Lu et al. (38)	Case-control study	35	ORS	MCHC	MCHC >347.5 g/L predicts the effect of ORS for treating POTS
Li et al. (39)	Case-control study	54	ORS	BMI	BMI <18 kg/m^2^ predicts the effect of ORS for treating POTS
Lin et al. (40)	Case-control study	34	Metoprolol	CNP	CNP >32.55 pg/m predicts the effect of metoprolol for treating POTS
Zhang et al. (41)	Case-control study	27	Metoprolol	Orthostatic plasma norepinephrine	Orthostatic plasma norepinephrine level of >3.59 pg/ml predicts the effect of metoprolol for treating POTS
Zhao et al. (42)	Case-control study	33	Midodrine	Copeptin	A plasma copeptin level >10.482 pmol/L predicts the effect of midodrine for treating POTS
Yang et al. (43)	Case-control study	28	Midodrine	Erythrocytic hydrogen sulfide	Erythrocytic hydrogen sulfide production rate >27.1 nmol/min/10^8^ erythrocytes predicts the effect of midodrine for treating POTS
Liao et al. (29)	Case-control study	108	Midodrine	FMD	FMD >9.85% predicts the effect of midodrine for treating POTS
Zhang et al. (44)	Case-control study	57	Midodrine	MR-proADM	The plasma concentration of MR-proADM >61.5 pg/ml predicts the effect of midodrine for treating POTS
Zhang et al. (27)	Case-control study	30	Midodrine	24-h urinary sodium excretion	The 24-h urinary sodium excretion <124 mmol predicts the effect of midodrine for treating POTS

*POTS, postural orthostatic tachycardia syndrome; ORS, oral rehydration solution; MCHC, mean corpuscular hemoglobin concentration; BMI, body mass index; CNP, C-type natriuretic peptide; FMD, flow-mediated vasodilation; MR-proADM, midregional fragment of pro-adrenomedullin*.

## Non-Pharmacological Treatments

Lowered water intake and shorter sleeping time were identified as POTS risk factors in children and adolescents (45). Health education is an important part of treatment of POTS patients. The basic treatment of the disease is to increase water and salt intake. Most children with POTS need have salt intake of up to 5–6 g. Urine osmolality of <300 mmol/L or 24-h urinary sodium excretion of more than 200 mmol are the goals (46). Good sleep may also be an important consideration, since we found that those getting <8 h of sleep per day were at 5.9 times greater risk of getting POTS than those with sleeping longer than 8 h (45).

Some investigators have found that a regular, short-term progressive physical-exercise program leads to improved symptoms in POTS patients (47). Such a regimen consists of rowing on a machine, swimming, recumbent and upright biking, and treadmill walking. However, one major challenge of successful physical therapy is patient compliance with regular and consistent with the program (6, 48).

Children and adolescents with POTS should be treated using a multidisciplinary approach that includes alternative nutritional, psychological, and drug therapies. Like other researchers, we have found that children with POTS have poor nutrition, including low iron storage (5, 49), vitamin B12 deficiency (50), vitamin B1 deficiency (51), hypovitaminosis D (52), and elevated plasma homocysteine levels (53). Correcting these would be very beneficial to the recovery of patients.

## Pharmacological Treatments

There is currently no pharmacologic therapy approved by the FDA for children with POTS. All the medications used to treat POTS in children are “off-label”, and most of the studies assessing POTS treatments are not evidence-based. There is lack of drug for POTS in multi-center randomized-control trials (RCT), and longitudinal long-term follow-up data of any medication for POTS are in need (6–9, 54). Wells et al. reported in a systemic review article that only 3 RCTs including 103 patients were studied for medications treatment of POTS in a single center (55). Our center reported that midodrine hydrochloride is effective in the treatment of children with POTS through a small non-blinded randomized controlled study (38). Drug therapy includes fludrocortisones for increasing central blood volume, the peripheral selective alpha-1-adrenergic agonist midodrine to constrict peripheral veins and reduce stagnant venous blood, β-adrenergic blockers, including non-selective propranolol, and cardioselective metoprolol, to decrease standing excessive tachycardia, and the acetylcholinesterase inhibitor pyridostigmine to increase acetylcholine on the autonomic ganglia, thereby enhancing ganglionic neurotransmission, increasing the release of norepinephrine by post-ganglionic sympathetic nerves and potentiating vagal effects upon standing (1, 4, 6, 8). However, the fact that “what works for one patient does not always work for another” was clearly displayed by this POTS patient, as Boris said (9). Wells et al. also pointed out that due to heterogeneity in the pathophysiology underlying POTS, biomarkers may play an incremental role in refining therapy (55). Based on our studies, POTS is a group of heterogeneous ailments caused by multiple sources of pathogenesis. If we could target drugs based on the pathogeneses of individual patients, the curative effects of drugs on children with POTS would be greatly enhanced. Each of the three major subtypes represents a different mechanism, and on this basis we have proposed an individualized-treatment strategy.

## Treatment of Hypovolemic Pots: Salt Supplements, Oral Rehydration and Fludrocortisone

Hypovolemia can be seen in many POTS patients, and increasing fluids intake is an effective treatment of POTS symptoms. A low concentration of urinary sodium is an indirect marker of hypovolemia (23, 27). Our previous work showed that, compared with the healthy control group, the concentration of 24-h urinary sodium was significantly lower in the POTS patients, and the symptom severity of children with POTS inversely correlated at a significant level with their 24-h urinary-sodium concentrations. Twenty-four hours sodium concentrations of <124 mmol/24 h indicated the effectiveness of salt supplements for treating POTS patients (sensitivity 76.9%; specificity 93%) (27).

Recent studies have found that using oral-rehydration solution (ORS) can conveniently, safely and effectively relieve the symptoms of children with POTS (56). Lu et al. found that among these children, those responding to ORS had a lower baseline mean-corpuscular volume (MCV) and higher mean-corpuscular hemoglobin concentration (MCHC) than non-responders, and that MCHC values are good predictors of ORS therapy for children and adolescents with POTS (39).

Stewart et al. found that, compared with POTS patients with normal blood volumes, the body-mass index (BMI) of POTS patients with hypovolemia was significantly lower. This finding suggests that BMI is related to blood volume (57). Our team found that BMI value lower than 18 kg/m^2^ indicated the effectiveness of ORS treatment for POTS children and adolescents (sensitivity 92%; specificity 82.8%). Compared with 24-h sodium concentrations, BMI is a simple and easily measured indicator (58).

Fludrocortisone is a synthetic mineralocorticoid that promotes the reabsorption of sodium and water in the kidneys. Therefore, it can increase plasma volume in patients with POTS (59–61). Theoretically, it can ameliorate the symptoms of hypovolemic POTS. However, compared with a placebo, fludrocortisone was found to be ineffective at relieving the symptoms of patients with neurally-mediated hypotension and chronic-fatigue syndrome in a placebo-controlled randomized trial (62). In another double-blind, placebo-controlled randomized trial, fludrocortisone also did not ameliorate the frequency of syncope in the treatment of vasovagal syncope patients (63). The reason that the drug has not been shown to be effective is non-selective use of it. Fludrocotisone should be used for hypovoleomic patients only. Also, attention should be paid to hypokalemia in the application of flurocortisoneone, since flurocortisone can promote the excretion of potassium. Fludrocortisone increases potassium excretion (59, 60). Fludrocortisone is now in the Heart Rhythm Society (HRS) Expert Consensus Class 2B recommendation for POTS patients (8).

## Treatment of Neuropathic Pots: Midodrine and Pyridostigmine

In neuropathic POTS, impaired peripheral vasoconstriction caused by adrenergic denervation can lead to peripheral venous pooling (64). Midodrine, an alpha1-adrenergic agonist, can effectively constrict peripheral vessels, and increase venous return. In a small non-blinded RCT, we found that midodrine could improve the symptoms of POTS in children and reduce standing heart rate (38). In order to select the drugs accurately, we seek biomarkers that reflect the presence of peripheral vasoconstriction dysfunction in POTS patients. FMD is an ultrasound technique used to assess blood-vessel elasticity (43). We found that, compared with the control children, baseline FMD increase was significantly greater in children with POTS, and the sensitivity and specificity of FMD at 9.85% as a cutoff value for predicting the short-term efficacy of midodrine-hydrochloride (1 month) treatment for POTS were 71.6 and 77.8%, respectively (29).

Hydrogen sulfide (H_2_S) is an important gaseous intracellular signal transducer. It is involved in the regulation of the cardiovascular system; it plays an important role in the pathogenesis of a variety of heart diseases, and is a novel gasotransmitter in the cardiovascular system (65). Our previous study demonstrated that erythrocytic H_2_S could indicate the effectiveness of midodrine hydrochloride for treating POTS patients. The sensitivity and specificity of erythrocytic H_2_S production of 27 nmol/min/10^8^ erythrocytes as a cutoff value for predicting the effectiveness of midodrine hydrochloride for children with POTS were 78.9 and 77.8%, respectively (44).

Adrenomedullin (ADM) is a potent vasodilator and has peripheral vasorelaxing effects. It is also associated with peripheral vasoconstriction and relaxation. But ADM has a short half-life, and easily adheres non-specifically to cellular surfaces, making its quantification impossible. Therefore, a midregional fragment of pro-adrenomedullin (MR-proADM), which produced quantities equivalent to those of ADM, is more stable than ADM (42). Zhang et al. found that the responders to midodrine hydrochloride in children with POTS had higher plasma levels of MR-proADM. MR-proADM >61.5 pg/ml predicts the efficacy of midodrine-hydrochloride therapy for treating POTS (sensitivity 100% and specificity 71.6%) (66).

Arginine vasopressin (AVP) plays an important role in circulatory and water homoeostasis. Copeptin and arginine vasopressin (AVP) are derived from a common precursor molecule and have equimolar secretion, and copeptin is more stable in plasma than AVP. Our group found that copeptin was also a good biomarker predicting the effectiveness of midodrine hydrochloride in treating children with POTS (67). Midodrine is now in an HRS Class 2B recommendation for the treatment of POTS (8).

Neuropathic POTS is regarded as a restricted autoimmune autonomic ganglionopathy (AAG), associated with auto-antibodies in the ganglionic acetylcholine receptor (30, 31). Along with others, we found that this type of POTS in children and adolescents was associated with positive acetylcholine receptor antibodies (AChR-ab) (16, 31). Pyridostigmine, a peripheral acetylcholinesterase inhibitor, could be used to treat patients with POTS. Its therapeutic mechanism involves increasing synaptic acetylcholine in the autonomic ganglia and functional enhancement of nerve conduction of parasympathetic nervous systems. It has also been shown that pyridostigmine can increase the baroreceptor sensitivity of POTS patients and thereby ameliorate their symptoms (68). In a randomized crossover study, Raj et al. found that pyridostigmine significantly attenuated tachycardia and ameliorated the symptoms of POTS, and in another long-term retrospective study they also found that pyridostigmine could improve the standing heart rate and ameliorated the symptoms of POTS (41, 68). Filler et al. reported that a 16-years-old girl with severe POTS was treated with pyridostigmine, and that after 9 months there were persistent positive effects without additional blood-pressure abnormalities (40). We propose that if a child with POTS is seropositive for AChR-ab, pyridostigmine should be used appropriately. It is in an HRS Class 2B recommendation for the treatment of POTS (8).

## Treatment of Hyperadrenergic Pots: β-Blockers

The characteristics of hyperadrenergic POTS is an elevated upright plasma norepinephrine levels. This subtype of patients with orthostatic hypertension has posed a great challenge to the traditional treatment of POTS (34, 35). Salt supplements and peripheral vasoconstrictor-midodrine should be used with caution in treating these patients. In the treatment of this type of POTS, β-blockers for blocking β-adrenoceptors are preferred, which could prevent the effect of having excessive catecholamines in the plasma. We found that the severity of symptoms and increments of the heart rate when standing was positively correlated with the plasma norepinephrine levels of patients in upright positions, and compared with non-responders to metoprolol, the upright plasma norepinephrine levels in responders to metoprolol were significantly high. The sensitivity and specificity of an orthostatic plasma-norepinephrine level of 3.59 mmol/ml as a cutoff value for predicting the effectiveness of metoprolol for children with POTS were 76.9 and 91.7%, respectively (69). However, plasma-norepinephrine levels were not stable in circulation, and were affected by many factors, including exercise and emotions. Thus, we should seek a stable, easily detected and inexpensive biomarker of hyperadrenergic POTS. C-type natriuretic peptide (CNP) is a regulatory peptide that can affect catecholamine release. Lin et al. reported that, compared with non-responders to metoprolol therapy of POTS in children and adolescents, plasma CNP in responders was significantly high before treatment, and baseline plasma CNP of >32.6 pg/ml predicted the efficacy of metoprolol therapy for treating POTS in children (sensitivity 100% and specificity 71.6%). Thus, plasma CNP is a useful clinical predictor of the therapeutic response to metoprolol in POTS patients (70).

Propranolol, a non-selective β-blocker, can pass the blood-brain barrier. Thus, it is considered to work better than other β-blockers in the treatment of POTS (71). Some studies have found that low doses of propranolol are superior to higher doses in the treatment of POTS adult patients. Low dosages of oral propranolol ameliorates the upright tachycardia and enhances the capacity for exercise of POTS patients (72). Propranolol is also used as a migraine prophylaxis medication (73), making it sometimes useful for treating patients with POTS co-morbid migraine. Propranolol is in an HRS Class 2B recommendation for treatment of POTS (8).

**Figure 1:**
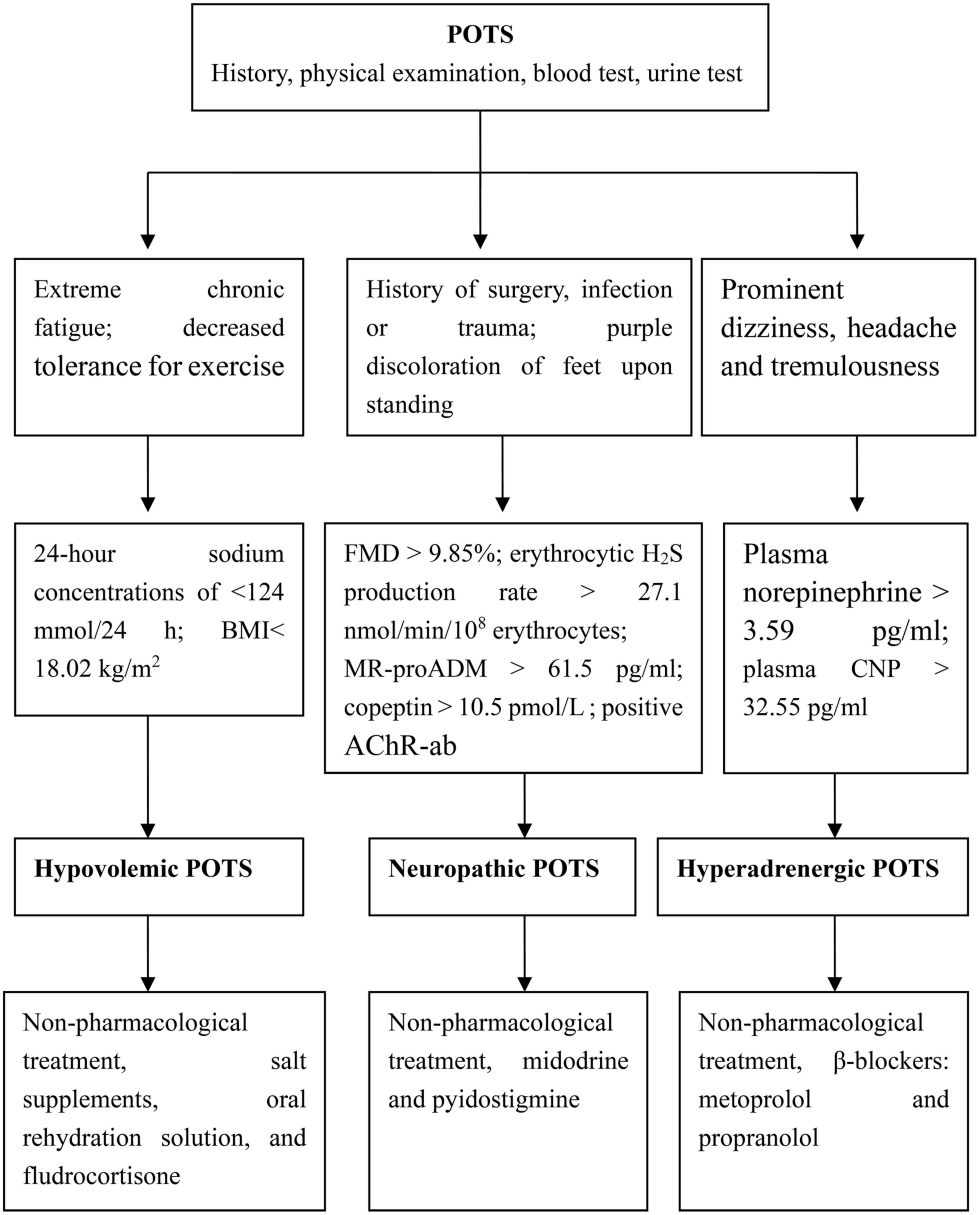
Individualized treatment strategies for children based on POT subtypes. POTS, postural orthostatic tachycardia syndrome; BMI, body-mass index; FMD, flow-mediated vasodilation; MR-proADM, mid-regional fragment of pro-adrenomedullin; AChR-ab, antibodies of acetylcholine receptor; CNP, C-type natriuretic peptide.

In summary, we have categorized the pathophysiology of POTS into 3 major subtypes that may overlap: hypovolemic, neuropathic and hyperadrenergic. It is important to note that strategies that work for some patients may not be applicable to all. Thus, in clinical practice, it is very important to search for biomarkers of the pathogenesis of POTS in children as a guide to select the drugs best suited to any given individuals. With this goal in mind, we have proposed a subtype pathophysiology-based personalized-treatment strategy (Figure 1).

## Conclusion

POTS is a common and heterogeneous disorder in children and adolescents that significantly reduces quality of life. Its common categories are hypovolemic, hyperadrenergic and neuropathic. Management always involves cross-disciplinary care that includes lifestyle changes, nutritional adjustments, exercise and drugs. Different sub-types of POTS have different clinical characteristics, and involve different physiological and biochemical changes. Some biomarkers reflect the pathogenesis of POTS in children and guide the choice of drugs for individualized treatment. However, a further clinical investigation of its pathophysiology and the options for treating it is still required.

## Author Contributions

QZ had primary responsibility for the design and execution of the study, data collection, preliminary data analysis, and writing the manuscript. BX participated in data collection, preliminary data analysis, and the writing of the manuscript. JD supervised the design and execution of the study, performed the final data analyses, and contributed to the writing of the manuscript.

## Conflict of Interest

The authors declare that the research was conducted in the absence of any commercial or financial relationships that could be construed as a potential conflict of interest.
